# MicroRNA Regulation in the Freeze-Tolerant Heart of *Dryophytes versicolor*

**DOI:** 10.3390/genes16090997

**Published:** 2025-08-25

**Authors:** Saif Rehman, Sarah A. Breedon, Imane Rhzali, Kenneth B. Storey

**Affiliations:** Institute of Biochemistry and Department of Biology, Carleton University, Ottawa, ON K1S 5B6, Canada; sarahbreedon@cmail.carleton.ca (S.A.B.); imanerhzali@cmail.carleton.ca (I.R.); kenstorey@cunet.carleton.ca (K.B.S.)

**Keywords:** *Dryophytes versicolor*, freeze tolerance, cardiac tissue, metabolic rate depression, sRNA-sequencing, miR-93-5p, let-7b-5p

## Abstract

**Background:** Freeze tolerance is an uncommon but highly effective strategy that allows certain vertebrates to survive prolonged exposure to subzero temperatures in a frozen, ischemic state. While past studies have characterized the metabolic and biochemical adaptations involved, including cryoprotectant accumulation and metabolic rate suppression, the contribution of post-transcriptional gene regulation by microRNAs (miRNAs) remains largely unexplored. This study investigated freeze-responsive miRNAs in cardiac tissue of the gray tree frog, *Dryophytes versicolor*, to better understand the molecular mechanisms that support ischemic survival and tissue preservation. **Methods:** Adult frogs were subjected to controlled freezing at −2.5 °C, and cardiac tissue was collected from frozen and control animals. Total RNA was extracted and analyzed via small RNA sequencing to identify differentially expressed miRNAs, followed by target gene prediction and KEGG pathway enrichment analysis. **Results:** A total of 3 miRNAs were differentially expressed during freezing, with significant upregulation of miR-93-5p and let-7b-5p and downregulation of miR-4485-3p. Predicted targets of upregulated miRNAs included genes involved in immune signaling pathways (e.g., cytokine–cytokine receptor interaction), steroid hormone biosynthesis, and neuroactive ligand–receptor interaction, suggesting suppression of energetically costly signaling processes. Downregulation of miRNAs targeting cell cycle, insulin signaling, and WNT pathways indicates possible selective preservation of cytoprotective and repair functions. **Conclusion:** Overall, these results suggest that *D. versicolor* employs miRNA-mediated regulatory networks to support metabolic suppression, maintain essential signaling, and prevent damage during prolonged cardiac arrest. This work expands our understanding of freeze tolerance at the molecular level and may offer insights into biomedical strategies for cryopreservation and ischemia–reperfusion injury.

## 1. Introduction

The grey tree frog, *D. versicolor*, is a frog species found in the Eastern United States and Southeastern Canada. The winter months in these regions can induce a variety of extreme environmental stresses including lack of food, anoxia, and cold—often subzero—temperatures. In order to withstand these harsh conditions, the grey tree frog, like many other stress-tolerant animals, has evolved mechanisms that allow for survival. One mechanism these animals use is freeze tolerance. Freeze tolerance is the ability to withstand ice formation in the extracellular spaces whilst also protecting the internal organs from freezing. *D. versicolor* can survive up to 60% of its total body water being turned into ice [1]. Therefore, cryoprotectants are essential for survival; one that many animals use including the grey tree frog is glycerol [2]. In the animal’s frozen state, their heart stops beating, their breathing ceases, and many regulatory functions stop until spring when they begin to thaw [3]. This means that the animal has to rely on anaerobic metabolism and stored fuel reserves as they can no longer produce energy at normal physiological levels [4]. However, to cope with the limited energy supply, the frogs must enter a state of metabolic rate depression (MRD). MRD is a mechanism that involves the global down regulation of nonessential genes [5]. While frozen, the consumption of ATP should not exceed the ATP production. However, because they are frozen, the frog’s ability to produce ATP is significantly reduced and they must rely on anaerobic glycolysis to meet their energy demands. Therefore, MRD is essential for survival as this ensures that energy-expensive processes are reduced, and any energy demands are to sustain survival. These animals use a variety of processes and mechanisms to regulate MRD, with one being microRNA-mediated suppression of gene expression [3].

Recent studies have shown the potential importance of microRNAs (miRNAs), which are short, noncoding, single-stranded RNA, in eliciting MRD. They are typically 18–25 nucleotides long and play an important role in the regulation of gene expression, specifically in eukaryotic cells. Many studies have shown the important role miRNAs play in biological processes, especially due to their conservation throughout evolutionary history, with many being shared among distantly related species. The primary role of miRNA’s is to regulate expression of genes via the inhibition of messenger RNA (mRNA) translation. They do this by binding to complementary sequences on the target mRNA; this can then result in either degradation or translational repression [6]. Furthermore, a single miRNA can target mRNA transcripts for multiple genes, or one mRNA can be affected by multiple miRNA species [7]. This further highlights the extensive roles that miRNAs play in regulating metabolic reorganization. There have been many studies in different animals subjected to a range of environmental stressors that have shown the importance of miRNA in regulating MRD and gene expression [8,9,10]. These findings highlight the crucial role of miRNAs in stress tolerance.

Given the broad involvement of miRNAs in regulating cellular pathways under normoxic conditions, it is strongly suggested that they do play a role in stress response. *D. versicolor* is capable of surviving prolonged periods of stress, making it an excellent model for this study. Previous studies have been done on the grey tree frog, and they observed changes in expression of miRNA specifically in the muscle and liver where energy-expensive pathways were downregulated [1,7]. This specific study will focus on the heart of the freeze-tolerant grey tree frog. The heart is crucial for survival and may utilize different mechanisms than those of the liver or muscle tissue to survive stress with minimal damage. Therefore, to gain a deeper understanding of the mechanisms that enable these animals to endure extreme stress, the present study performed small RNA-seq analysis on control and 24 h frozen grey tree frog cardiac tissue. This study aims to explore the role that miRNA expression may play in MRD, specifically in the cardiac tissue of the freeze-tolerant *D. versicolor*. The identification and understanding of the regulatory changes in the frozen grey tree frog can offer insights into crucial adaptations required for surviving extreme environmental stress.

## 2. Results

### 2.1. Small RNA Sequencing Summary

**Figure 1:**
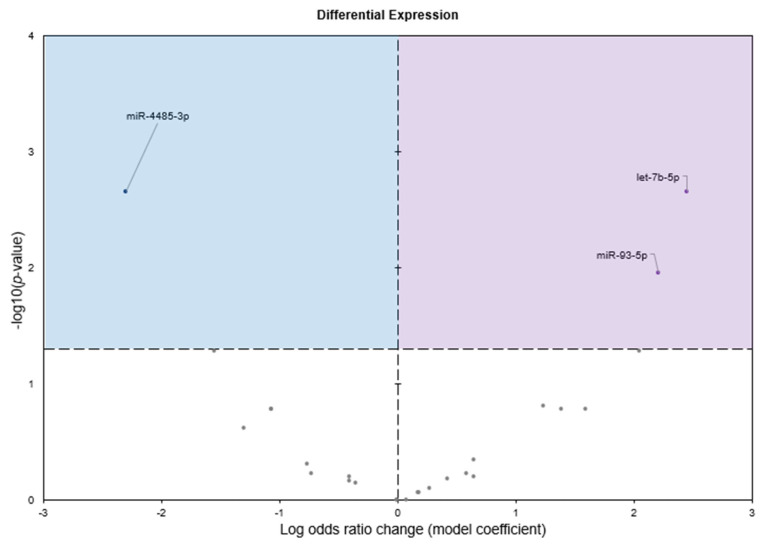
Differential expression analysis of conserved miRNAs in heart muscle tissue of *D. versicolor* (*n* = 3). Volcano plot of the differential expression of conserved miRNAs showing −log_10_(*p* value) versus the log_2_(fold change) for individual miRNAs in heart muscle from frozen frogs versus the control group. miRNAs are considered significantly altered if the FDR (False Discovery Rate) adjusted *p*-value is <0.05 and fold change >1.5. Significantly upregulated miRNAs are depicted on the right of the plot as purple circles, whereas miRNAs that are significantly downregulated are depicted on the left by blue circles.

Raw sequencing reads averaged 36,870,086 ± 1,974,929 and 66,904,835 ± 13,732,427 in control and frozen *D. versicolor* hearts, respectively. Following all filtering steps, there were 12,051,873 ± 1,182,343 and 19,070,955 ± 3,251,352 reads that aligned to 27 known mature miRNAs in control and freezing, respectively. Of the mature miRNAs identified, three miRNAs were significantly differentially expressed in freezing; two were upregulated in freezing (let-7b-5p, miR-93-5p), whereas one was downregulated (miR-4485-3p; Figure 1).

### 2.2. Gene Set Analyses

**Figure 2:**
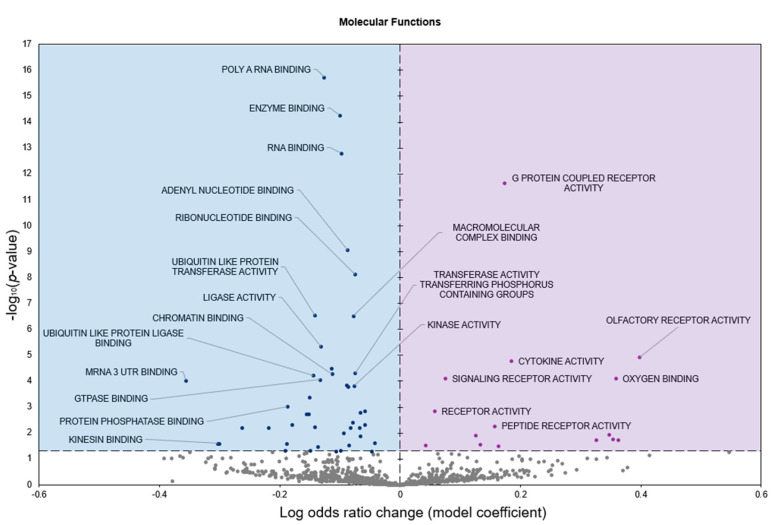
Volcano plot representing GO molecular function (GO MF) gene set analysis of the mRNAs predicted to interact with differentially expressed (DE) miRNAs in *n* = 3 *D. versicolor* heart in response to freezing. Positive model coefficients (**right**) depicting increased expression are indicated by purple circles, whereas negative model coefficients (**left**) depicting decreased expression are shown by blue circles.

Gene Ontology molecular functions (GO MF) analysis predicted 15 terms to be significantly enriched in response to freezing with positive model coefficients, whereas 45 terms had negative model coefficients (*p* < 0.05; Figure 2).

**Figure 3:**
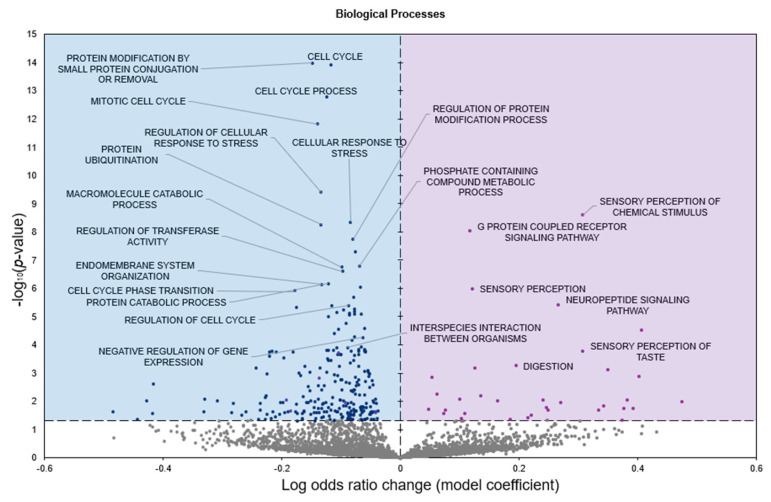
Volcano plot representing GO biological processes (GO BP) gene set analysis of the mRNAs predicted to interact with DE miRNAs in *n* = 3 *D. versicolor* heart in response to freezing. All other information as in Figure 2.

GO biological processes (GO BP) analysis identified 34 terms predicted to be significantly enriched in response to freezing with positive model coefficients, whereas 250 terms had negative model coefficients (*p* < 0.05; Figure 3).

**Figure 4:**
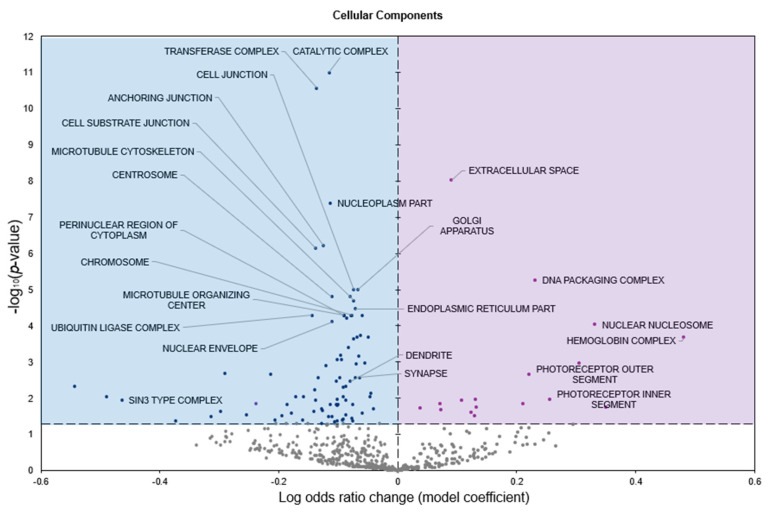
Volcano plot representing GO Cellular Components (GO CC) gene set analysis of the mRNAs predicted to interact with DE miRNAs in *n* = 3 *D. versicolor* heart in response to freezing. All other information as in Figure 2.

GO cellular components (GO CC) analysis predicted 17 terms to be significantly enriched in response to freezing with positive model coefficients, whereas 83 terms had negative model coefficients (*p* < 0.05; Figure 4).

**Figure 5:**
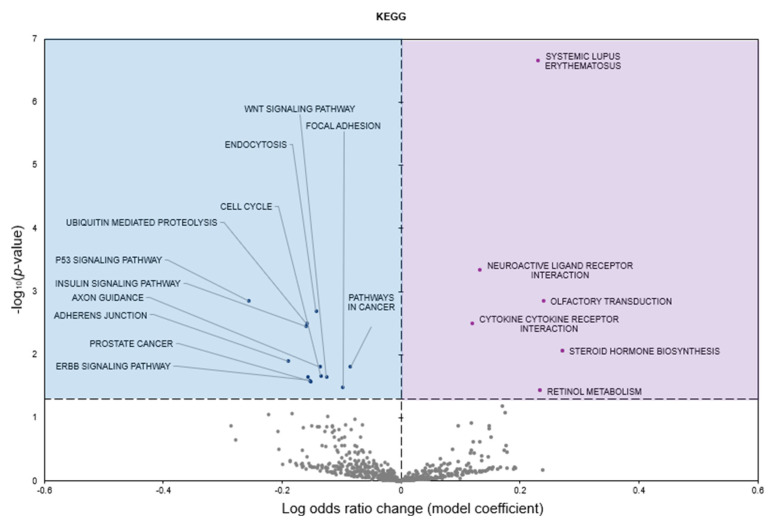
Volcano plot representing KEGG pathway gene set analysis of the mRNAs predicted to interact with DE miRNAs in *n* = 3 *D. versicolor* heart in response to freezing. All other information as in Figure 2.

Kyoto Encyclopedia of Genes and Genomes (KEGG) analysis predicted six terms to be significantly enriched in response to freezing with positive model coefficients, whereas 13 terms had negative model coefficients (*p* < 0.05; Figure 5).

## 3. Discussion

Freeze tolerance in *D. versicolor* represents a rare and highly specialized physiological adaptation that enables survival under extreme environmental stress. This process necessitates precise molecular regulation to mitigate the damaging effects of prolonged ischemia, osmotic stress, and oxidative damage while maintaining tissue integrity for rapid post-thaw recovery. The heart plays a central role in freeze tolerance, as it must withstand prolonged cessation of circulation and rapidly resume function upon thawing. Our study identifies significant upregulation of miR-93-5p and let-7b-5p, along with downregulation of miR-4485-3p, in cardiac tissue during freezing (Figure 1). Functional enrichment analysis of differentially expressed miRNAs revealed key pathways associated with immune modulation, metabolic control, and cellular stress responses, all of which are integral to freeze tolerance (Figure 2, Figure 3 and Figure 4). These findings suggest a cardiac-specific regulatory network that governs freeze tolerance in *D. versicolor*, providing valuable insights into the molecular strategies underlying vertebrate freeze tolerance and potential biomedical applications. Our findings suggest that miRNA-mediated regulation of key cardioprotective, metabolic, and structural pathways is a crucial component of this process.

The upregulation of miR-93-5p suggests a critical role in protecting cardiac endothelial cells, reducing oxidative stress, and promoting vascular remodeling under ischemic conditions. This miRNA is known to regulate Phosphatase and TENsin homolog deleted on chromosome 10 (PTEN) and Vascular Endothelial Growth Factor (VEGF) signaling, which influence angiogenesis and vascular integrity, both of which are essential for cardiac tissue survival during freezing-induced ischemia [11,12]. In *D. versicolor*, miR-93-5p may contribute to maintaining endothelial stability and preventing vascular collapse, ensuring efficient reperfusion upon thawing. Additionally, its regulation of insulin signaling and energy metabolism pathways suggests a role in metabolic suppression, a well-established strategy in freeze-tolerant and hibernating species to conserve ATP and prevent excessive reactive oxygen species (ROS) production [13]. Similarly, let-7b-5p is a key regulator of stress adaptation, metabolic suppression, and cellular quiescence, making it highly relevant in cardiac freeze tolerance. The let-7 family regulates metabolic rate suppression by targeting Insulin-like Growth Factor 1 Receptor (IGF1R) and (MYC), reducing unnecessary energy expenditure and promoting hypometabolism [14,15,16,17]. Upregulation of let-7b-5p in frozen cardiac tissue suggests an active suppression of growth and proliferation, aligning with previous findings in hibernators and aestivators [18,19,20]. This regulatory shift ensures that the heart maintains structural integrity without undergoing energy-intensive cellular remodeling, allowing it to withstand freezing and resume function efficiently upon thawing. The downregulation of miR-4485-3p suggests an essential role in apoptosis inhibition and mitochondrial stability during freezing. miR-4485-3p has been linked to p53-mediated apoptosis and mitochondrial dysfunction, and its suppression is associated with enhanced cell survival under ischemic stress [21]. In *D. versicolor*, reduced expression of miR-4485-3p may prevent excessive apoptotic signaling in cardiomyocytes, helping to preserve heart tissue during prolonged ischemia.

Enrichment analysis of upregulated KEGG pathways supports the upregulation of pathways involved in cardioprotective roles. The systemic lupus erythematosus pathway, which involves histone modifications and chromatin remodeling, may facilitate the broad transcriptional reprogramming required for freeze tolerance [22,23]. Additionally, cytokine-cytokine receptor interactions suggest an adaptive immune response in cardiac tissue, which may prevent excessive inflammatory damage upon thawing [24,25]. Increased activity in steroid hormone biosynthesis and retinol metabolism suggests that cardiac tissue undergoes lipid-based membrane stabilization and antioxidant defense, mechanisms known to be protective against ischemia–reperfusion injury. Furthermore, activation of neuroactive ligand–receptor interaction and olfactory transduction pathways suggests that neural and sensory regulation may contribute to cardiac autonomic control during freezing.

KEGG pathway analysis of downregulated miRNAs further supports this cardioprotective function (Figure 5). Suppression of pathways such as WNT signaling, focal adhesion, p53 signaling, and insulin signaling suggests an adaptive shift toward metabolic suppression and cytoskeletal stabilization, reducing unnecessary cellular activity during freezing [3,26,27]. The inhibition of focal adhesion and adherens junction pathways indicates a temporary reduction in cytoskeletal remodeling [10,28], which may help stabilize cardiomyocytes against freeze-induced mechanical stress. Similarly, suppression of p53 signaling suggests that the heart actively downregulates apoptotic pathways to enhance cardiomyocyte survival, mirroring strategies observed in hibernating mammals. Additionally, reduced activity in the insulin signaling pathway aligns with the known metabolic suppression observed in freeze-tolerant species [3,29,30,31]. Insulin signaling is closely linked to glucose metabolism, and its suppression suggests a shift toward alternative fuel utilization, such as glycogen mobilization and lipid metabolism, which are crucial for maintaining energy balance during prolonged freezing. The downregulation of pathways in cancer and ErbB signaling suggests a global suppression of cellular proliferation [32], further reinforcing the idea that *D. versicolor* cardiac tissue enters a reversible state of quiescence during freezing.

While freeze tolerance is a systemic adaptation, the heart faces unique challenges due to its role in circulation, oxygen delivery, and ischemic resistance. The miRNA-mediated regulatory shifts observed in this study suggest that *D. versicolor* cardiac tissue undergoes a carefully coordinated transition into a hypometabolic, stress-resistant state, ensuring its survival and rapid functional restoration upon thawing. Upregulated miRNAs appear to promote vascular protection, metabolic suppression, and immune modulation, while the downregulated miRNA contributes to apoptosis inhibition, reduced cytoskeletal remodeling, and overall energy conservation. Comparison of the current dataset with previous *D. versicolor* studies in liver and skeletal muscle indicates that while the identities of freeze-responsive miRNAs differ between tissues, the predicted functional outcomes converge on similar regulatory themes. In liver, eleven miRNAs change significantly during freezing, with enrichment patterns suggesting suppression of signaling, apoptotic, and nuclear functions coupled with increased ribosomal biogenesis to support selective translation under energy-limited conditions [7]. In skeletal muscle, five miRNAs are differentially expressed, and predicted targets indicate inhibition of cell cycle progression and metabolic activity, while maintaining capacity for essential signaling processes [33]. Together with the current cardiac data, these results support a model in which distinct tissue-specific miRNA repertoires act within a shared framework to promote metabolic suppression, preserve structural integrity, and enhance stress resilience during freezing. Comparable functional themes are also observed in the thirteen-lined ground squirrel heart during hibernation, where miRNA changes are highly tissue- and stage-specific yet consistently predict repression of growth and proliferative processes alongside cytoprotective signaling [34]. This cross-taxa consistency suggests that post-transcriptional regulation by miRNAs may represent a broadly conserved mechanism for coordinating hypometabolism and ischemia tolerance across freeze-tolerant amphibians and mammalian hibernators. These findings provide insights into the molecular underpinnings of vertebrate freeze tolerance and highlight potential biomedical applications, such as strategies for ischemia–reperfusion injury mitigation, organ preservation, and therapeutic approaches for cardiac ischemic diseases.

## 4. Materials and Methods

### 4.1. Animal Experiments

Adult male *D. versicolor* frogs weighing approximately 6–10 g were collected from Ottawa area (Ottawa, ON, Canada) breeding ponds and treated as previously described [33]. All animal protocols followed approved Canadian Council on Animal Care guidelines and were approved by the Carleton University Animal Care Committee (protocol no. 13683). Briefly, all frogs were washed with tetracycline and acclimated for 2 weeks in boxes with damp sphagnum moss and no food at 5 °C. Following acclimation, control frogs (*n* = 3) were sampled, and the remaining frogs (*n* = 3) were moved to trays with damp paper towel and placed in −4 °C incubator. After the initial ice nucleation was triggered in the frogs, the temperature of the incubator was raised to −2.5 °C for 24 h to allow the frogs to fully freeze. Control and frozen frogs were euthanized via double-pithing, and hearts were excised and flash frozen in liquid nitrogen before being stored at −80 °C until use.

### 4.2. Small RNA Sequencing

*D. versicolor* heart samples (*n* = 3 individual frogs each for control and frozen stress) were sent to Canada’s Michael Smith Genome Sciences Centre (Vancouver, BC, Canada), where RNA was extracted and small RNA sequencing was performed. RNA quality was validated using an Agilent Bioanalyzer 2100 (Agilent Technologies, Santa Clara, CA, USA) prior to and after construction of the miRNA library as previously described [10]. Sequencing was performed using an Illumina NextSeq2000 (San Diego, CA, USA), and all raw data is available on Sequence Read Archive (SRA; BioProject ID: PRJNA1236686).

### 4.3. Read Processing

Raw sequencing reads were analyzed using RBioMIR (https://github.com/jzhangc/git_RBioMIR, accessed on 8 January 2025) [35]. Briefly, cutadapt (version 0.10.1, https://cutadapt.readthedocs.io/en/stable/, accessed on 10 January 2025) was used to filter out low-quality reads and adapter sequences, with read quality/length analyzed before and after filtering using FastQC (http://www.bioinformatics.babraham.ac.uk/projects/fastqc/, accessed on 10 January 2025) [36] Bowtie (http://bowtie.cbcb.umd.edu accessed on 10 January 2025) was then used to remove non-miRNA small RNAs (tRNA, rRNA, piRNA, snRNA, and snoRNA) by comparison with the entirety of the Rfam and piRNABank databases [37,38]. Known mature miRNA sequences were then selected from the remaining reads using a positive reference file of all sequences from miRBase using a seed length of 20 nt [39]. Lastly, these mature miRNA reads were sorted and counted using samtools (https://www.htslib.org/, accessed on 10 January 2025), where miRNAs with less than four reads were removed from the dataset and the voom method was used for normalization [40].

### 4.4. Differential Expression Analysis

Differential miRNA expression of control and frozen frogs was determined as previously described [41]. The limma R package (version #3.64.3, https://bioconductor.org/packages/release/bioc/html/limma.html, accessed on 10 January 2025) was used with linear model fitting and empirical Bayesian testing [42]. Only differentially expressed miRNAs with an FDR-adjusted *p*-value < 0.05 and fold-change (FC) > 1.5 were accepted as statistically significant.

### 4.5. Gene Set Analysis

RBiomirGS (https://github.com/jzhangc/git_RBiomirGS, accessed on 10 January 2025) was used for GO and KEGG gene set analyses as previously described [43,44]. Briefly, miRNA scores (*S*_microRNA_) and mRNA scores (*S*_mRNA_) were calculated using miRNA–mRNA interactions to predict significantly enriched gene sets and calculate GO and KEGG term model coefficients. Positive model coefficients indicate predicted upregulation of the term due to decreased negative miRNA-mediated regulation, whereas negative model coefficients indicate predicted term downregulation due to increased negative miRNA-mediated regulation in frozen frogs relative to control frogs.

## 5. Conclusions

Our findings suggest that *D. versicolor* employs miRNA-mediated regulation to balance stress resilience, metabolic suppression, and tissue preservation in the heart during freezing. The upregulation of miRNAs associated with vascular protection and immune modulation, combined with the suppression of apoptotic and metabolic pathways, highlights a complex but highly coordinated cardioprotective strategy. Given the predictive nature of these data, future studies should focus on validating the miRNA–mRNA target interactions identified herein. Additionally, assessing protein-level regulatory changes and investigating tissue-specific miRNA dynamics would aid in fully elucidating the mechanisms underlying cardiac freeze tolerance. Moreover, exploring the potential for miRNA-based interventions in ischemic heart disease and cryopreservation could open new avenues for translational research.

## Data Availability

All sequencing data is available on Sequence Read Archive (SRA; BioProject ID: PRJNA1236686). The data that support the findings of this study are available from the corresponding author upon reasonable request.

## Abbreviations

The following abbreviations are used in this manuscript:

miRNA	MicroRNA
MRD	Metabolic Rate Depression
GO	Gene Ontology
KEGG	Kyoto Encyclopedia of Genes and Genomes
